# Supplementary material for: Decrease in α-Globin and Increase in the Autophagy-Activating Kinase ULK1 mRNA in Erythroid Precursors from β-Thalassemia Patients Treated with Sirolimus
Source: Int J Mol Sci. 2023 Oct 10;24(20):15049. doi: 10.3390/ijms242015049 (PMC10606773; doi:10.3390/ijms242015049)
Supplement: Supplementary file 1 [file ijms-24-15049-s001.zip › ijms-2613948-Supplementary.pdf]

Article

Decrease in α-Globin and Increase in the Autophagy-Activating Kinase ULK1 mRNA in Erythroid Precursors from β-Thalassemia Patients Treated with Sirolimus

Matteo Zurlo [1], Cristina Zuccato [1,2], Lucia Carmela Cosenza [1], Jessica Gasparello [1], Maria Rita Gamberini [3], Alice Stievano [3], Monica Fortini [3], Marco Prosdocimi [4], Alessia Finotti [1,2,*] and Roberto Gambari [1,2,*]

[1] Department of Life Sciences and Biotechnology, Ferrara University, 44121 Ferrara, Italy; matteo.zurlo@unife.it (M.Z.); cristina.zuccato@unife.it (C.Z.); luciacarmela.cosenza@unife.it (L.C.C.); jessica.gasparello@unife.it (J.G.)
[2] Center "Chiara Gemmo and Elio Zago" for the Research on Thalassemia, Ferrara University, 44121 Ferrara, Italy
[3] Thalassemia Unit, Arcispedale S. Anna, 44121 Ferrara, Italy; gamberinimariarita@gmail.com (M.R.G.); stievano.alice@gmail.com (A.S.); monica.fortini@ospfe.it (M.F.)
[4] Rare Partners S.r.L. Impresa Sociale, 20123 Milano, Italy; m.prosdocimi@rarepartners.org
* Correspondence: alessia.finotti@unife.it (A.F.); gam@unife.it (R.G.)

SUPPLEMENTARY MATERIAL

Supplementary Figures

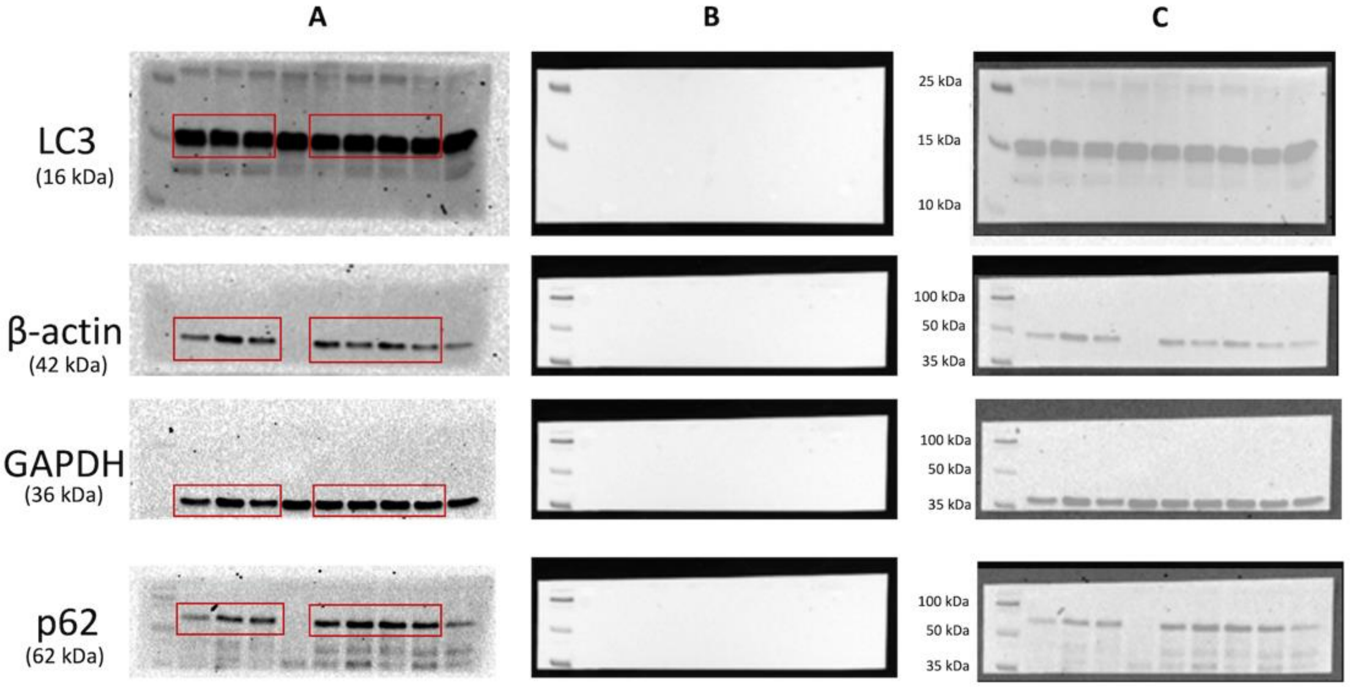

Figure S1. Uncropped version of Western Blot presented in Figure 1A. In panel A we show the acquired blot image, in panel B the nitrocellulose membrane with the prestained multicolor protein ladder (Spectra pre-stained ladder by Thermo Fisher, Waltham, MA, USA, cat. n. 26634) and in panel C the merge of picture A and B, showing the exact molecular weight of the target proteins.

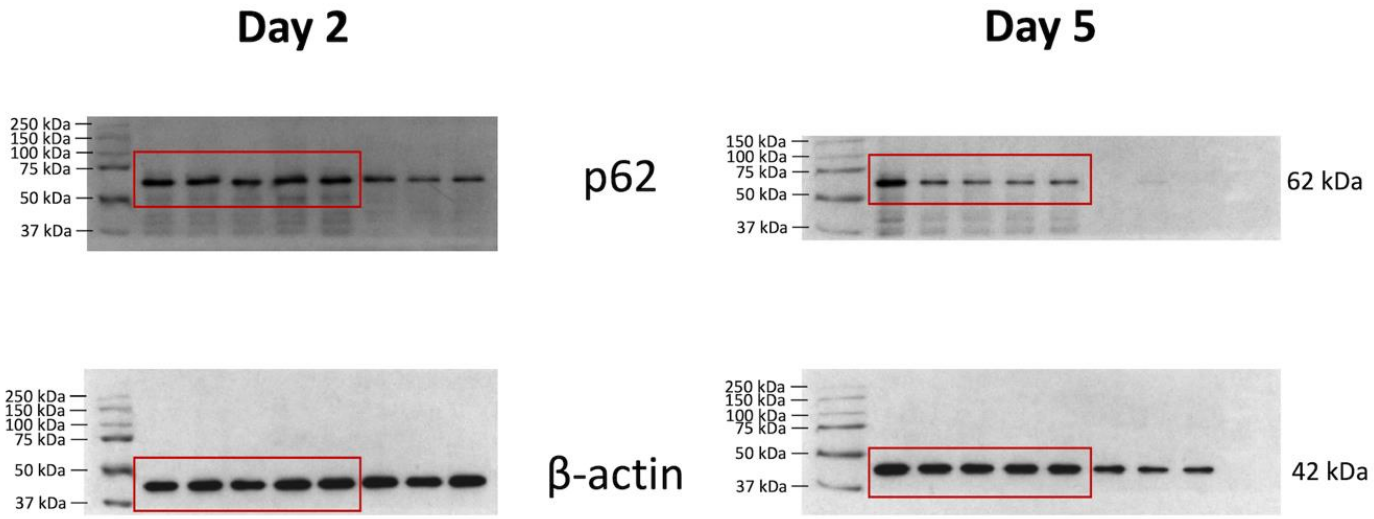

Figure S2. Uncropped version of Western Blot presented in Figure 2A. On the left of each picture we clearly show the molecular weight corresponding to each band of the protein ladder employed (Precision Plus Protein WesternC Standard from Bio-Rad, Hercules, CA, USA, cat. n. 1610376).

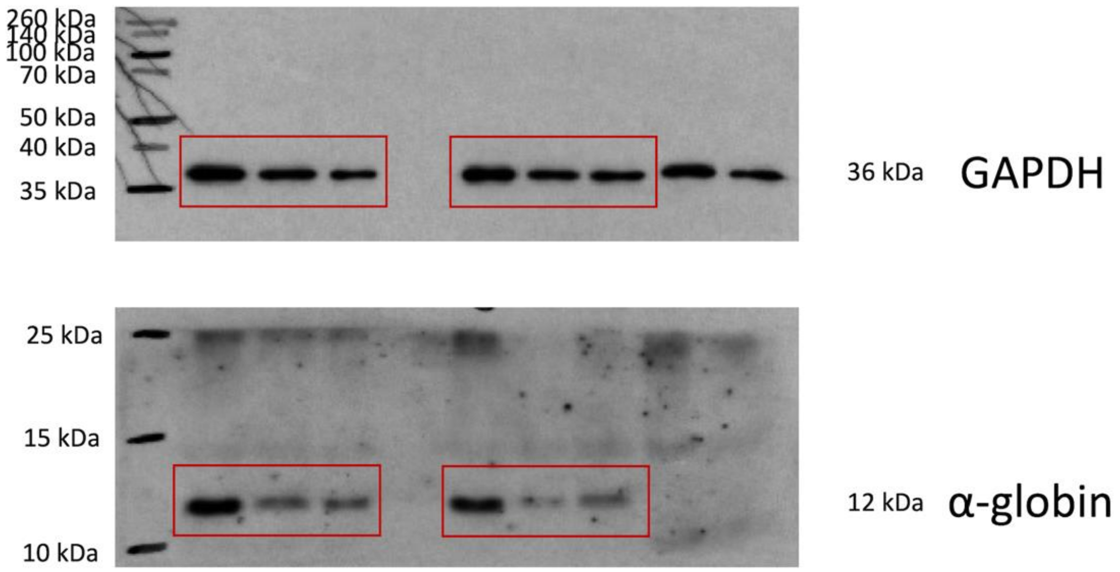

Figure S3. Uncropped version of Western Blot presented in Figure 3A. On the left of each picture we clearly show the molecular weight corresponding to each band of the protein ladder employed (Spectra pre-stained ladder by Thermo Fisher, Waltham, MA, USA, cat. n. 26634).
